# Minor loading vein acclimation for three *Arabidopsis thaliana* ecotypes in response to growth under different temperature and light regimes

**DOI:** 10.3389/fpls.2013.00240

**Published:** 2013-07-05

**Authors:** Christopher M. Cohu, Onno Muller, Barbara Demmig-Adams, William W. Adams

**Affiliations:** Department of Ecology and Evolutionary Biology, University of ColoradoBoulder, CO, USA

**Keywords:** *Arabidopsis thaliana* ecotypes, leaf vasculature, light acclimation, minor veins, phloem, temperature acclimation

## Abstract

In light of the important role of foliar phloem as the nexus between energy acquisition through photosynthesis and distribution of the products of photosynthesis to the rest of the plant, as well as communication between the whole plant and its leaves, we examined whether foliar minor loading veins in three *Arabidopsis thaliana* ecotypes undergo acclimation to the growth environment. As a winter annual exhibiting higher rates of photosynthesis in response to cooler vs. warmer temperatures, this species might be expected to adjust the structure of its phloem to accommodate greater fluxes of sugars in response to growth at low temperature. Minor (fourth- and third-order) veins had 14 or fewer sieve elements and phloem tissue comprised 50% or more of the cross-sectional area. The number of phloem cells per minor loading vein was greater in leaves grown under cool temperature and high light vs. warm temperature and moderate light. This effect was greatest in an ecotype from Sweden, in which growth under cool temperature and high light resulted in minor veins with an even greater emphasis on phloem (50% more phloem cells with more than 100% greater cross-sectional area of phloem) compared to growth under warm temperature and moderate light. Likewise, the number of sieve elements per minor vein increased linearly with growth temperature under moderate light, almost doubling over a 27°C temperature range (21°C leaf temperature range) in the Swedish ecotype. Increased emphasis on cells involved in sugar loading and transport may be critical for maintaining sugar export from leaves of an overwintering annual such as *A. thaliana*, and particularly for the ecotype from the northern-most population experiencing the lowest temperatures.

## Introduction

Mature green leaves, through the process of photosynthesis, produce sugars in excess of the leaves' own metabolic needs and export large quantities of sugars to the many sinks of the plant. It is well-known that plant sink activity, in turn, feeds back on photosynthesis, with high sink activity inducing upregulation of photosynthesis and the genes underlying photosynthetic activity and low sink activity acting to repress photosynthetic genes and photosynthesis (Layne and Flore, 1993; Paul and Foyer, 2001). Recent studies have furthermore suggested that the process of loading sugars into the plant's sugar-transporting phloem for export from the mature leaf (phloem loading) may also be tied tightly into the leaf's photosynthetic performance. The leaf's rate of photosynthesis may be influenced, or even limited, by phloem features and phloem loading in the leaf's minor loading veins (Amiard et al., 2005; Adams et al., 2007, 2013; Ainsworth and Bush, 2011; Nikinmaa et al., 2013).

Insect vectors that tap into loading veins can furthermore introduce various pathogens that utilize the phloem to travel to other portions of the leaf and throughout the plant (Ding et al., 1995, 1998; Gilbertson and Lucas, 1996; Lucas and Wolf, 1999; Cheng et al., 2000; Heller and Gierth, 2001; Zhou et al., 2002; Taliansky et al., 2003; Waigman et al., 2004; Scholthof, 2005; Rasheed et al., 2006; Requena et al., 2006; Saha et al., 2006; Gosálvez-Bernal et al., 2008; Peter et al., 2009; Vuorinen et al., 2011). Perhaps not surprisingly, the minor loading veins are responsive to not only pathogenic attack (Rioux and Quellette, 1991; Kpemoua et al., 1996; Narváez-Vásquez and Ryan, 2004; Narváez-Vásquez et al., 2005) but also to various signaling pathways involving reactive oxygen, antioxidants, and plant hormones (Provencher et al., 2001; Hofius et al., 2004; Maeda et al., 2006; Amiard et al., 2007; Scarpella et al., 2010; Wenzel et al., 2012; Baylis et al., 2013; Demmig-Adams et al., 2013; Zhiponova et al., 2013). Furthermore, the plant vascular conduits provided by phloem and xylem are primary avenues for communication among different parts of the plant (Turnbull and Lopez-Cobollo, 2013). As the nexus for leaf functioning and interaction with the rest of the plant, foliar vasculature plays a central role in many key processes.

The smallest foliar veins are therefore of broad interest to biologists working on many aspects of plant biology, including photosynthesis, signaling (e.g., redox signaling) networks, and biotic defense, and additional studies are needed to better understand the role of minor loading veins in plant metabolism and response to the environment. Such studies depend on an understanding of what distinguishes foliar minor loading veins (Esau, 1977) from larger veins. As a leaf develops, the phloem of the larger veins provide a passage for the import of carbohydrates into the developing (sink) leaves, with the minor veins only becoming functional in phloem loading and carbohydrate export once the leaf gains photosynthetic competency and transitions from being a sink to an active source of carbohydrates for the rest of the plant (Turgeon, 1989; McGarry and Ayer, 2008).

Haritatos et al. (2000) conducted a detailed characterization of the minor loading veins of the model plant *Arabidopsis thaliana* (Columbia ecotype) grown under a single controlled growth regime consisting of a 12 h photoperiod (photon flux density, or PFD, of only 200 μmol photons m^−2^ s^−1^) with 20°C/18°C day/night temperatures (leaf temperatures not specified). Given that *A. thaliana* is a winter-active annual species that upregulates photosynthesis in response to growth at lower temperatures (Strand et al., 1997, 1999), we suspected that it might also exhibit acclimatory adjustments in the phloem to accompany such photosynthetic acclimation. We therefore grew three ecotypes of *A. thaliana* under various environmental conditions to identify features of the phloem that may respond to those conditions.

## Materials and methods

### Plant species

Three *A. thaliana* (L.) Heynhold ecotypes were characterized, i.e., wildtype Columbia (Col-0; Arabidopsis Information Resource collection; http://www.arabidopsis.org/) and two ecotypes obtained from natural populations in north-central Sweden and central Italy (Ågren and Schemske, 2012). Species other than *A. thaliana* characterized for comparison include squash (*Cucurbita pepo* L. cv. Italian Zucchini Romanesco), sunflower (*Helianthus annuus* L. var. Soraya), and spinach (*Spinacia oleracea* L. cv. Giant Nobel).

### Growth conditions

*Arabidopsis thaliana* plants were grown from seed, after vernalization at 4°C for 4 days, under four different controlled growth-chamber conditions (leaf temperature of 24–26°C day/20°C night resulting from air temperatures of 25°C day/20°C night, or leaf temperature of 12–16°C day/12.5°C night resulting from air temperatures of 8°C day/12.5°C night, 9 h photoperiod of 400 or 1000 μmol photons m^−2^ s^−1^) where they were fertilized with nutrients every other day. Squash, sunflower, and spinach were grown from seed under controlled growth-chamber conditions (leaf temperature of 24–26°C day/20°C night resulting from air temperatures of 25°C day/20°C night, 9 h photoperiod of 400 μmol photons m^−2^ s^−1^). Mean daytime leaf temperatures of 25°C and 14°C are used throughout to refer to the respective growth temperature regimes. Plants grown at 14°C (8°C air temperature) were germinated at an air temperature of 25°C until cotyledons emerged, then transferred to 15°C (air temperature) for 1 week before transfer to 8°C (air temperature). Only fully expanded mature leaves of non-flowering *A. thaliana* plants (6–8 weeks old) that emerged under final growth conditions were characterized. *Arabidopsis thaliana* rosettes were 12–15 cm in diameter, and leaf blades characterized ranged between 3 and 5 cm in length (not including petiole). Only fully expanded, mature leaves of squash, sunflower, and spinach were characterized (4-week-old plants).

*Arabidopsis thaliana* plants were also grown under two additional temperature regimes (15°C day/18°C night and 35°C day/25°C night) under a 9 h photoperiod of 400 μmol photons m^−2^ s^−1^ for anatomical characterization.

### Leaf tissue preparation and vein measurements

During excision and glutaraldehyde fixation of tissue from fully expanded *A. thaliana* leaves, 2 mm^2^ segments were removed from regions between second-order veins (as defined by Hickey, 1973) for characterization of third- and fourth-order veins. Care was taken to sample tissue greater than 2 mm from the midrib and 3 mm from the leaf margin in the central region of the leaves (excluding the leaf apical and basal regions). Second-order veins were also characterized to (i) ensure that only third- and fourth-order veins, and not smaller portions or branches of second-order veins, were included in the analyses and (ii) determine whether any additional differences existed between second-order veins vs. third- and fourth-order veins. For squash, sunflower, and spinach plants, leaf tissue was excised as described for *A. thaliana* except that third-order veins in squash and sunflower were avoided because of their large size, resulting in a noticeable rib above and below the vein, and high vein order number present (seven vein orders for squash and sunflower). Leaf tissue embedding in Spurr resin for microscopy and tissue preparation for vein density measurements were conducted as described in Amiard et al. (2005). Tissue sections of 0.8 to1.0 μm for light microscopy were stained for 30 min (Toluidine blue, 0.1%; sodium borate, 1%; in water).

Seven to ten third- and fourth-order veins were characterized from each of four plants from each *A. thaliana* ecotype for each growth condition. All third- and fourth-order veins cut perpendicular to the vein were measured and included in analyses regardless of size. While vein order assignment as third- and fourth-order veins was straightforward for many veins, a clear differentiation between third- and fourth-order veins at a cross-sectional level was not possible, and third- and fourth-order veins were thus not segregated. Vein cross-sectional areas were measured using Image-J (Rasband W.S., ImageJ, U.S. National Institute of Health, Bethesda, Maryland, USA, http://imagej.nih.gov/ij/, 1997–2012), and linear regressions, correlation coefficients, and polynomial fit lines were created using JMP statistical software (SAS Institute, Cary, North Carolina). Comparison of regression lines (slope and intercept) was conducted using an analysis of covariance (ANCOVA) (Sokal and Rohlf, 1981). Comparisons among two means were conducted using a Student's *t*-test.

## Results

**Figure 1:**
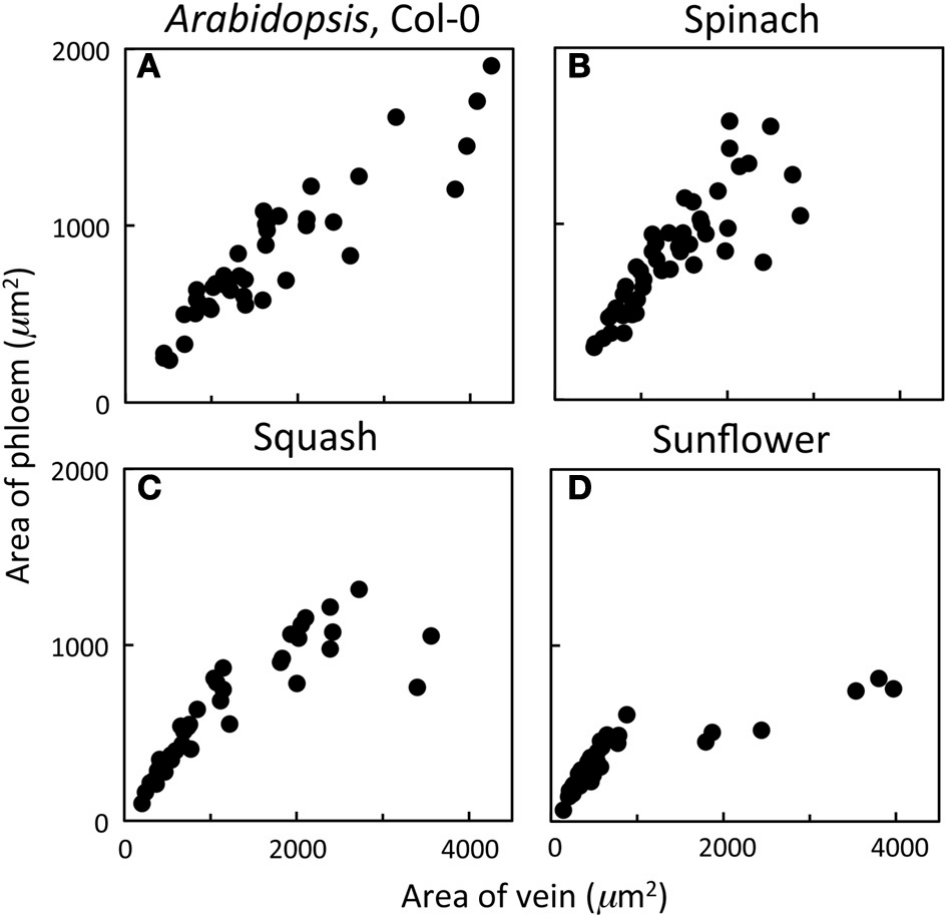
**Relationship between the cross-sectional area of phloem (all phloem cells combined) and the cross-sectional area of the entire vein for individual veins of (A) *A. thaliana* Col-0 (*n* = 44), (B) spinach (*n* = 45), (C) squash (*n* = 48), and (D) sunflower (*n* = 65) grown at 25°C under 400 μmol photons m^−2^ s^−1^**.

Phloem cross-sectional area of leaf sections (consisting of all sieve elements, companion cells, and phloem parenchyma cells bounded by the bundle sheath and xylem cells) increased in close proportion with increases in vein cross-sectional area for veins up to 2000 μm^2^ and exhibited a broader range of phloem areas for veins with areas greater than 2000 μm^2^ in leaves of *A. thaliana* Col-0, spinach, and squash grown at 25°C under 400 μmol photons m^−2^ s^−1^ (Figures 1A–C). On the other hand, sunflower grown under the same conditions possessed veins that exhibited proportional increases in phloem area with vein area only up to a vein area of 1000 μm^2^, no veins with cross-sectional areas between 1000 and almost 2000 μm^2^, and larger veins (2000–4000 μm^2^) showing little increase in phloem area (Figure 1D). This leveling off was therefore due to further substantial increases in xylem cross-sectional area with little increase in phloem cross-sectional area. Thus, classification of minor veins vs. larger veins, for which the increase in vein size is due largely to increased water-conducting xylem tissue, is a straightforward matter of measuring vein cross-sectional area in sunflower leaves, whereas a more detailed analysis of vein structure is required for distinguishing minor loading veins from larger veins in other species. A detailed analysis of the foliar veins of *A. thaliana*, and acclimatory adjustments of the phloem within the minor veins in response to different growth conditions, is presented here.

**Figure 2:**
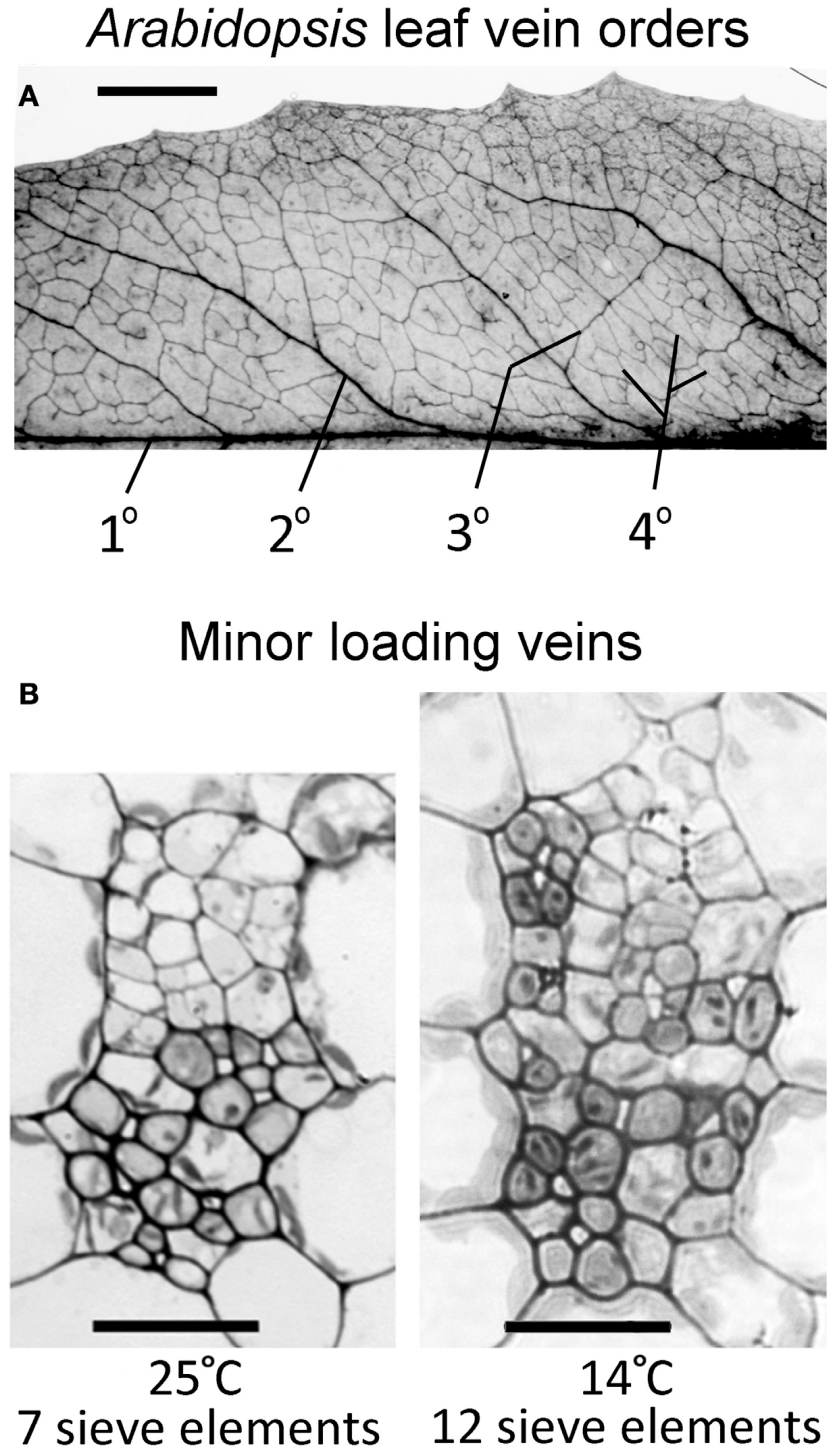
**(A)** Vein orders in a cleared *A. thaliana* leaf (Swedish ecotype) grown at 25°C, with first order (1°) being the midrib and second-order veins (2°) comprised of main vascular branches off the midrib (typically five per leaf side, with five shown here either as portions or in their entirety), as defined by Hickey (1973). Third-order veins (3°) branch off the second order veins, and fourth-order veins (4°) are the smallest and can branch off all vein orders. (**B**) Cross-sectional light microscopic images are representative of fourth-order veins from mature leaves grown in warm (25°C) or cool (14°C) conditions under moderate light (400 μmol photons m^−2^ s^−1^). The vein shown from the plant grown at 25°C (**B**, left panel) contains seven sieve elements in the lower (abaxial) phloem region of the vein and features phloem parenchyma (angular and light colored) and companion cells (rounded and dark colored). The vein shown from the plant grown at 14°C (**B**, right panel) contains 12 sieve elements and features associated phloem cells encroaching into the upper (adaxial) region occupied by xylem cells. Scale bars shown are 5 mm for vein pattern and order (**A**) and 20 μm for fourth-order veins (**B**, same scale for both panels). The Italian and Col-0 ecotypes exhibited the same venation pattern and similar vein cross-sectional ultrastructure as shown here for the Swedish ecotype.

Vasculature of mature, fully expanded *A. thaliana* leaves has been separated into four vein orders based on size and branching patterns (Hickey, 1973; Haritatos et al., 2000). The midrib is a first-order, or primary, vein, with 5 second-order veins branching off the midrib per leaf side (Figure 2A). Both first- and second-order veins exhibited distinct ribs above and below the vein, and a larger cross-sectional area and different phloem cell organization compared to that of third- and fourth-order veins (images not shown). Third- and fourth-order veins branched off a higher-order vein for all *A. thaliana* ecotypes and growth conditions characterized, as was also reported by Haritatos et al. (2000) for the *A. thaliana* Columbia ecotype. Second-, third-, and fourth-order veins indicated in Figure 2A are examples of regions selected for vascular cross-sectional characterization.

**Figure 3:**
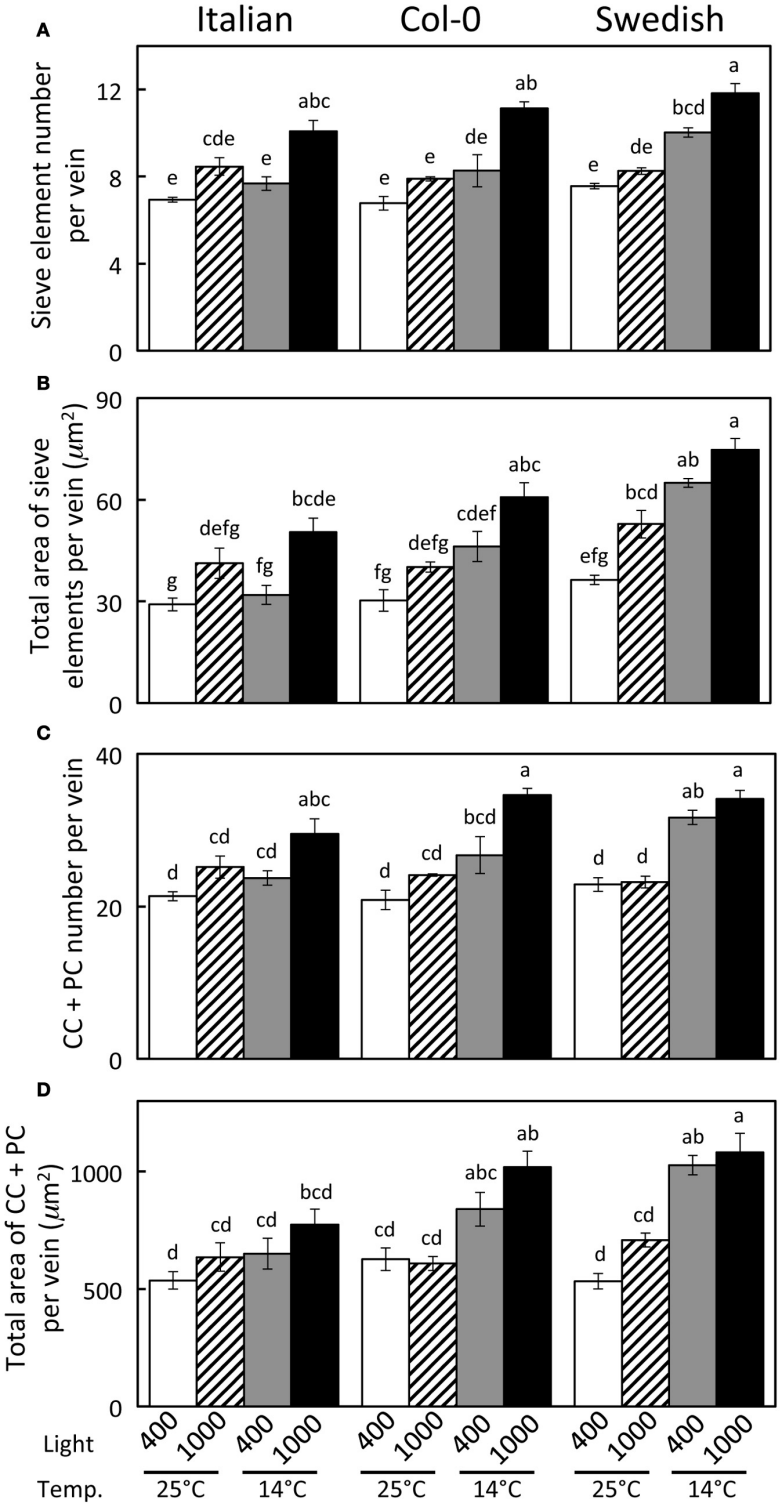
**Characterization of fourth- and third-order veins (veins containing ≤14 sieve elements and phloem area/vein area ≥0.5) from leaves of Italian, Col-0, and Swedish ecotypes of *A. thaliana* grown under different controlled temperature (°C daytime leaf temperature) and light (μmol photons m^−2^ s^−1^) conditions**. (**A**) Sieve element number per minor loading vein. (**B**) Cross-sectional area of sieve elements per minor loading vein. (**C**) Companion and phloem parenchyma cell (CC + PC) number per minor loading vein. (**D**) Cross-sectional area of CCs + PCs per minor loading vein. Mean ± standard error of the mean (*n* = 4 plants). Statistically significant differences indicated with lower case letters (*P* < 0.05), i.e., means sharing a common letter are not statistically different from one another.

**Figure 4:**
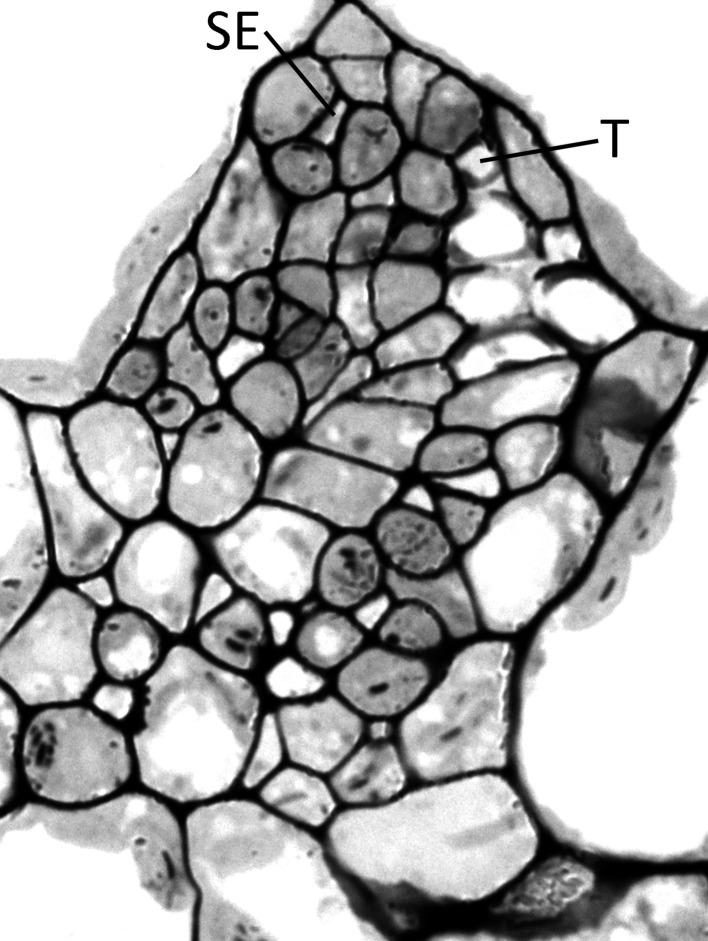
**Cross-section of a minor loading vein from the Swedish ecotype of *A. thaliana* grown under cool (14°C leaf temperature) and high light (1000 μmol photons m^−2^ s^−1^) conditions, illustrating the presence of phloem tissue beside and above the tracheids**. This vein possesses 13 sieve elements and 6 tracheids, with SE and T indicating the uppermost sieve element and tracheid, respectively.

Haritatos et al. (2000) used the number of sieve elements (sugar-transporting phloem cells) per vein to delineate vein order, with 10 or fewer sieve elements used as a good indicator of putative minor loading veins in *A. thaliana*. For all three ecotypes grown at 25°C under moderate or high light, all third- and fourth-order veins indeed contained 10 or fewer sieve elements (e.g., Figure 2B; number of sieve elements per minor vein ranged between 3 and 10). Compared to growth at 25°C under 400 μmol photons m^−2^ s^−1^, the number of sieve elements, and the total cross-sectional area occupied by sieve elements, was greater in third- and fourth-order veins under all other growth conditions (Figures 2B, 3A,B). Some third- and fourth-order veins of all ecotypes contained more than 10 (up to 14) sieve elements under high light and lower temperature (Figures 3A, 4). There was also a trend for a greater number, and greater cross-sectional area, of companion and phloem parenchyma cells with growth at lower temperature and at higher PFD, which was especially pronounced in the Col-0 and Swedish ecotypes (Figures 3C,D, 4). In addition, growth at lower temperature and greater PFD resulted in an altered organization of cell types within veins. All three *A. thaliana* ecotypes grown at 25°C exhibited a clear separation into upper (adaxial) xylem cells and lower (abaxial) phloem cells (Figure 2B, left panel). Plants grown at 14°C, however, did not always exhibit a clear delineation between regions of phloem and xylem cells, especially in the smaller fourth-order veins, although cell types were typically still discernable (Figure 2B, right panel). In leaves grown at 14°C, phloem cells were sometimes present in the lateral regions of the xylem (Figure 2B, right panel), and in rare cases were observed above (adaxial to) tracheids (the conducting elements of the xylem through which water and nutrients are transported) (Figure 4). In some cases, the number of xylem parenchyma cells was diminished.

Since Haritatos et al. (2000) had reported that minor loading (third- and fourth-order) veins likely possess no more than 10 sieve elements, and phloem structural changes in response to environmental conditions had not been reported previously, we conducted a detailed analysis of the structure and composition of over 425 veins from the three ecotypes of *A. thaliana* grown under various temperature and light regimes to ascertain that we were not inadvertently including second-order veins in our analyses. We used two criteria to compare minor loading veins with larger veins. One criterion was based on the finding of up to 14 sieve elements in the minor loading veins of the Swedish ecotype. A second feature was based of the proportion of the vein occupied by phloem cells, quantified as the cross-sectional area of the vein comprised of phloem tissue. Clearly discernible second-order veins of 14°C grown plants exhibited a cross-sectional area of phloem tissue relative to total vein area of 48% and less, while third and fourth order veins invariably exhibited a 57% or greater fraction of vein area comprised by phloem for the Swedish ecotype. Veins of *A. thaliana* were therefore classified as (i) minor loading veins when the number of sieve elements was ≤14 and phloem cross-sectional area constituted 50% or more of total cross-sectional area of the vein or (ii) larger second-order veins when the phloem contained 15 or more sieve elements and vein cross-sectional area occupied by phloem tissue constituted less than 50% of total cross-sectional area of the vein.

**Figure 5:**
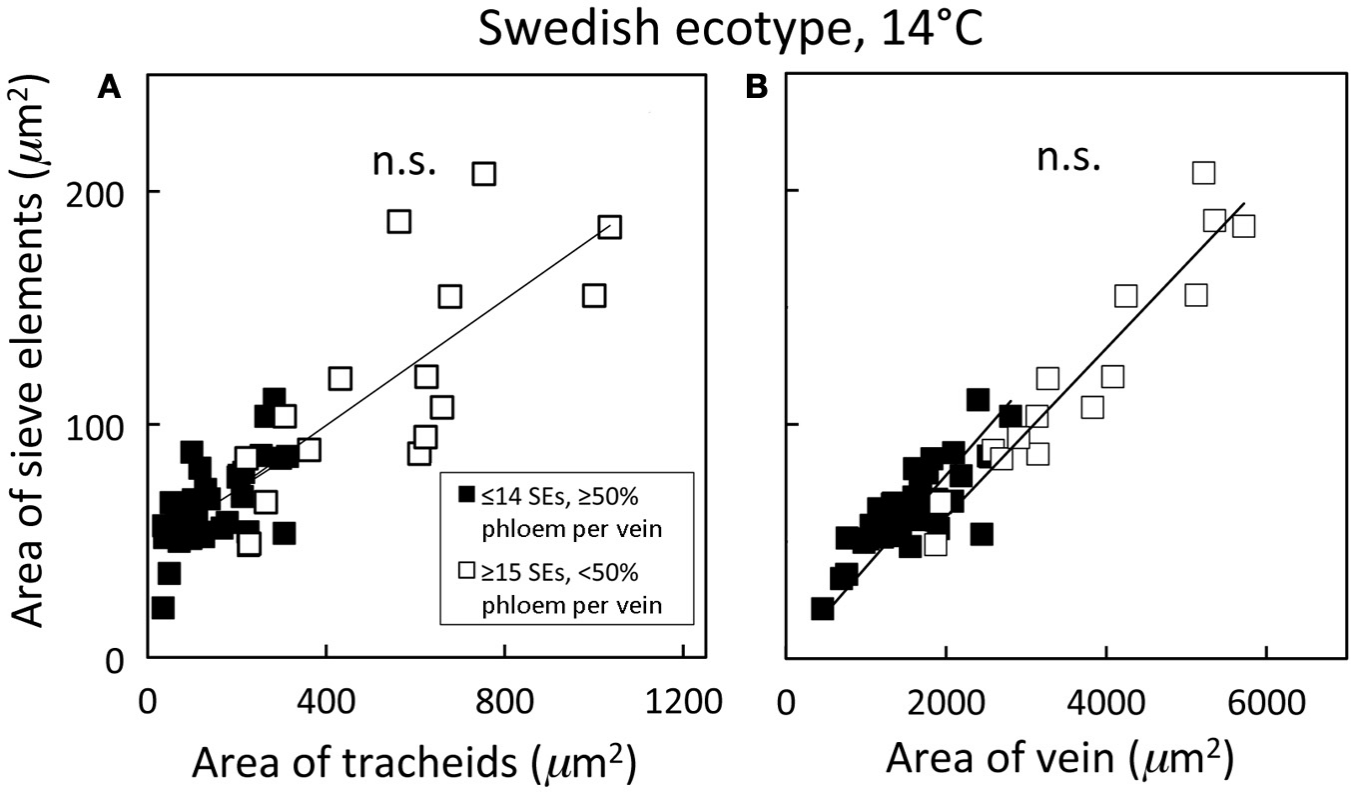
**Relationship between the cross-sectional sieve element area of a vein and (A) the cross-sectional tracheid area of a vein and (B) the cross-sectional area of the entire vein for individual second-, third-, and fourth-order veins from the Swedish ecotype grown at 14°C under 400 μmol photons m^−2^ s^−1^**. Individual veins (*n* = 52) were segregated by sieve element (SE) number and ratio of phloem area/vein area into veins containing ≤14 SEs and phloem area/vein area ≥0.5 (black squares; putative minor loading veins) or ≥15 SEs and phloem area/vein area <0.5 (open squares; second order veins). Linear regression lines drawn for each segregated group of veins (≤14 SEs or ≥15 SEs), and statistically significant differences in slope and intercept (ANCOVA) between regression lines is indicated (n.s. = not significant at *P* ≤ 0.05). Linear regression analyses for each line are (**A**) *y* = 0.12 *x* + 47, *R*^2^ = 0.34, and *P* < 0.01 (black squares) and *y* = 0.14 *x* + 46, *R*^2^ = 0.54, and *P* < 0.01 (open squares); (**B**) *y* = 0.03 *x* + 24, *R*^2^ = 0.64, and *P* < 0.001 (black squares) and *y* = 0.04 *x* − 11, *R*^2^ = 0.91, and *P* < 0.001 (open squares).

**Figure 6:**
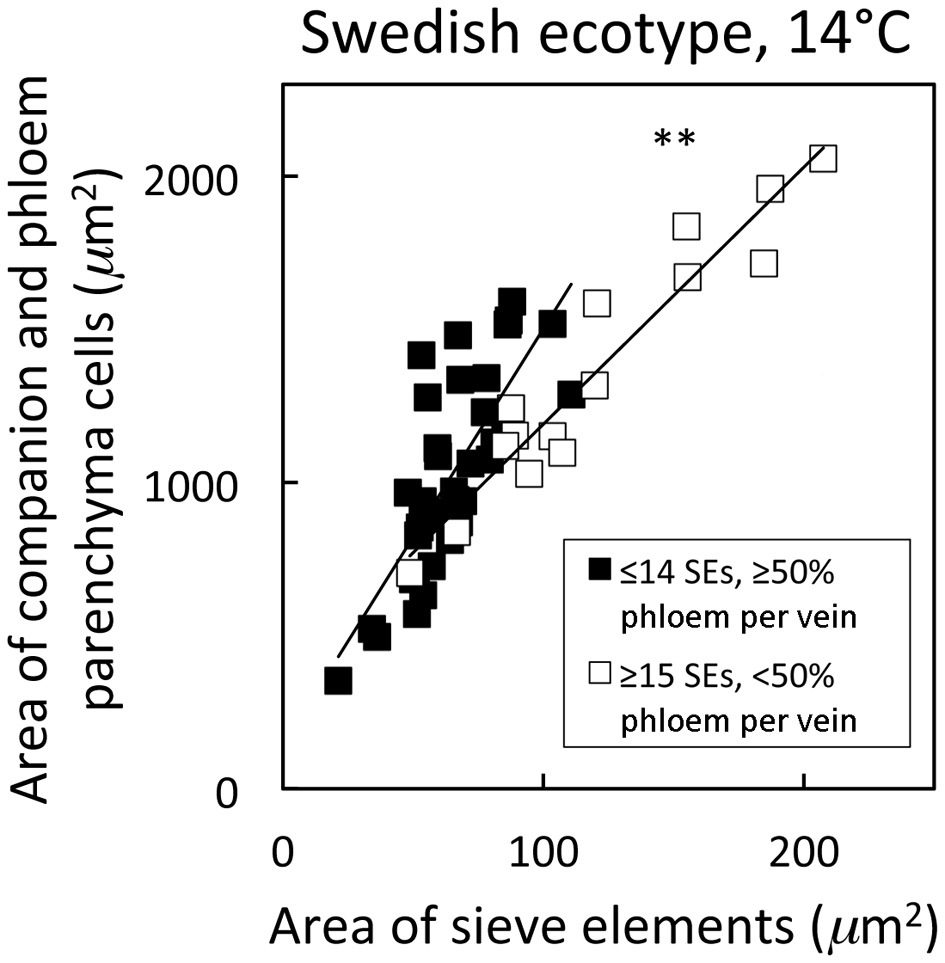
**Relationship between the cross-sectional area of companion and phloem parenchyma cells associated with sieve elements and the area of sieve elements for individual second-, third-, and fourth-order veins from the *A. thaliana* Swedish ecotype grown at 14°C under 400 μmol photons m^−2^ s^−1^**. Individual veins (*n* = 52) were segregated by sieve element (SE) number and ratio of phloem area/vein area into veins containing ≤14 SEs and phloem area/vein area ≥0.5 (black squares; putative minor loading veins) or ≥15 SEs and phloem area/vein area <0.5 (open squares; second order veins). Linear regression lines drawn for each segregated group of veins (≤14 SEs or ≥15 SEs), and statistically significant differences in slope and intercept (ANCOVA) between regression lines is indicated (^**^ = *P* < 0.01). Linear regression analyses for each line are *y* = 13.6 *x* + 142, *R*^2^ = 0.60, and *P* < 0.001 (black squares) and *y* = 8.4 *x* + 351, *R*^2^ = 0.91, and *P* < 0.001 (open squares).

**Figure 7:**
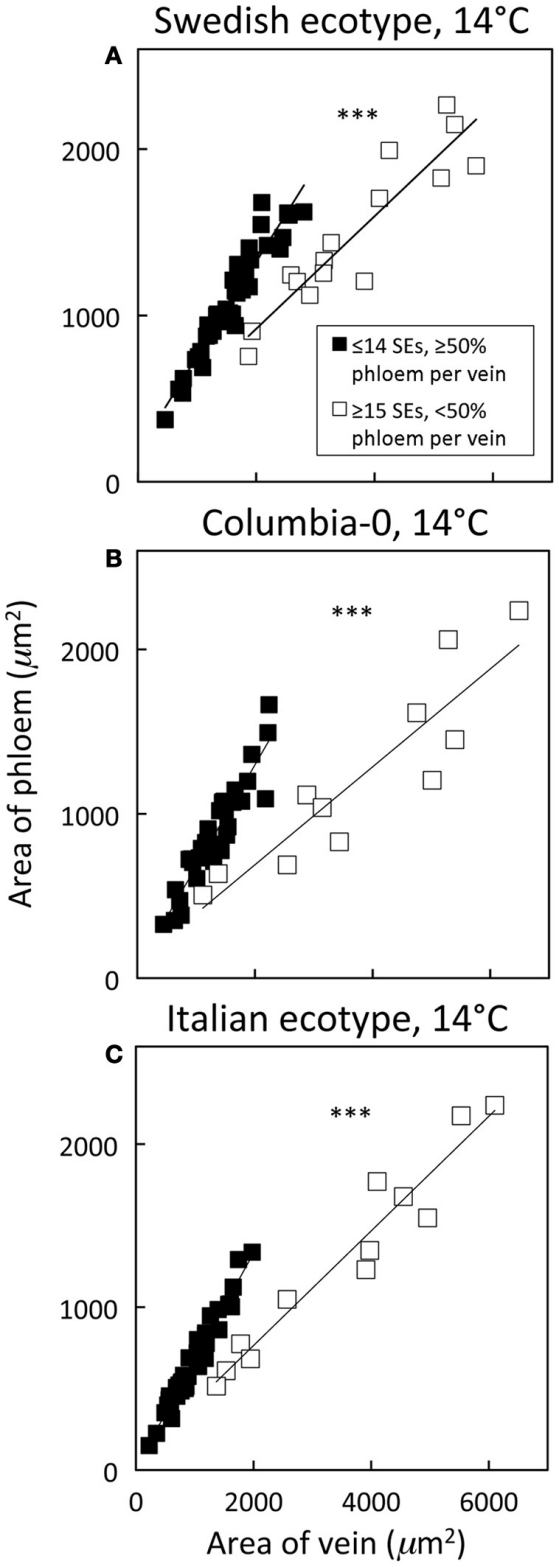
**Relationship between phloem cross-sectional area (all phloem cells combined) and total vein cross-sectional area for individual second-, third-, and fourth-order veins from the *A. thaliana* Swedish (A), Col-0 (B), and Italian (C) ecotypes grown at 14°C under 400 μmol photons m^−2^ s^−1^**. Individual veins (*n* = 52, Swedish; *n* = 56, Italian; *n* = 46, Col-0) were segregated by sieve element (SE) number and ratio of phloem area/vein area into veins containing ≤14 SEs and phloem area/vein area ≥0.5 (black squares; putative minor loading veins) or ≥15 SEs and phloem area/vein area <0.5 (open squares; second order veins). Linear regression lines shown for each segregated group of veins (≤14 SEs or ≥15 SEs), and statistically significant differences in slope and intercept (ANCOVA) between regression lines is indicated (^***^ = *P* < 0.001). Linear regression analyses for each line are (**A**) *y* = 0.57 *x* + 184, *R*^2^ = 0.91, and *P* < 0.001 (black squares) and *y* = 0.34 *x* + 243, *R*^2^ = 0.85, and *P* < 0.001 (open squares), (**B**) *y* = 0.62 *x* + 60, *R*^2^ = 0.87, and *P* < 0.001 (black squares) and *y* = 0.30 *x* + 94, *R*^2^ = 0.82, and *P* < 0.001 (open squares), and (**C**) *y* = 0.66 *x* + 15, *R*^2^ = 0.96, and *P* < 0.001 (black squares) and *y* = 0.35 *x* + 60, *R*^2^ = 0.94, and *P* < 0.001 (open squares).

Distinguishing second-order veins from third- and fourth-order veins of the Swedish ecotype of *A. thaliana* grown under 14°C and moderate light of 400 μmol photons m^−2^ s^−1^ as described above revealed several clear patterns. As one might expect, cross-sectional area of sieve elements of the phloem increased proportionally with increases in the area of tracheids of the xylem as veins increased in size from fourth- and third-order to larger second-order veins (Figure 5A). Likewise, total cross-sectional area of sieve elements per vein increased in proportion to total cross-sectional vein area over the range of veins characterized (Figure 5B), as would be expected to allow sugar flow from fourth- and third-order tributaries into larger second-order veins. On the other hand, third- and fourth-order veins possessed a considerably greater cross-sectional area of those phloem cells (companion cells and phloem parenchyma cells) directly associated with sieve elements when compared with veins classified as larger, second-order veins, although the smallest of the second-order veins did overlap with the minor loading veins (Figure 6). An allocation of a greater amount of tissue to companion and phloem parenchyma cells involved in moving sucrose into the sieve elements in fourth- and third-order veins would indeed be consistent with the role of these latter veins in actively loading sugars, and this trend is continued into the stem tissue where companion cells are greatly diminished compared to sieve elements (Esau, 1977). Moreover, second-order veins could be separated completely from third- and fourth-order veins when cross-sectional area of all phloem cells (sieve elements + companion cells + phloem parenchyma cells) was plotted against total cross-sectional vein area (Figure 7). Fourth- and third-order veins thus, once again, clearly exhibited a greater emphasis on phloem for a given cross-sectional vein area than the larger second-order veins for all three *A. thaliana* ecotypes (Figure 7).

In the present analysis, a small number of putative third- and fourth-order veins (from plants grown at cool temperature) that contained 14 or fewer sieve elements, but did not contain at least 50% of their vein cross-sectional area dedicated to phloem, were not included here as minor loading veins. One possible reason for this anomaly is that these veins may have been from second-order vein branches off the main second-order vein; such occasional branching or forking in second-order veins has been described (Hickey, 1973). An example for such an infrequently encountered branch may be the upward branch coming off the labeled second-order vein in Figure 2A (for another example, see Haritatos et al., 2000). Inclusion of such smaller-than-usual second-order veins is not likely to skew data in *A*. *thaliana* due to the low abundance of such branches.

**Figure 8:**
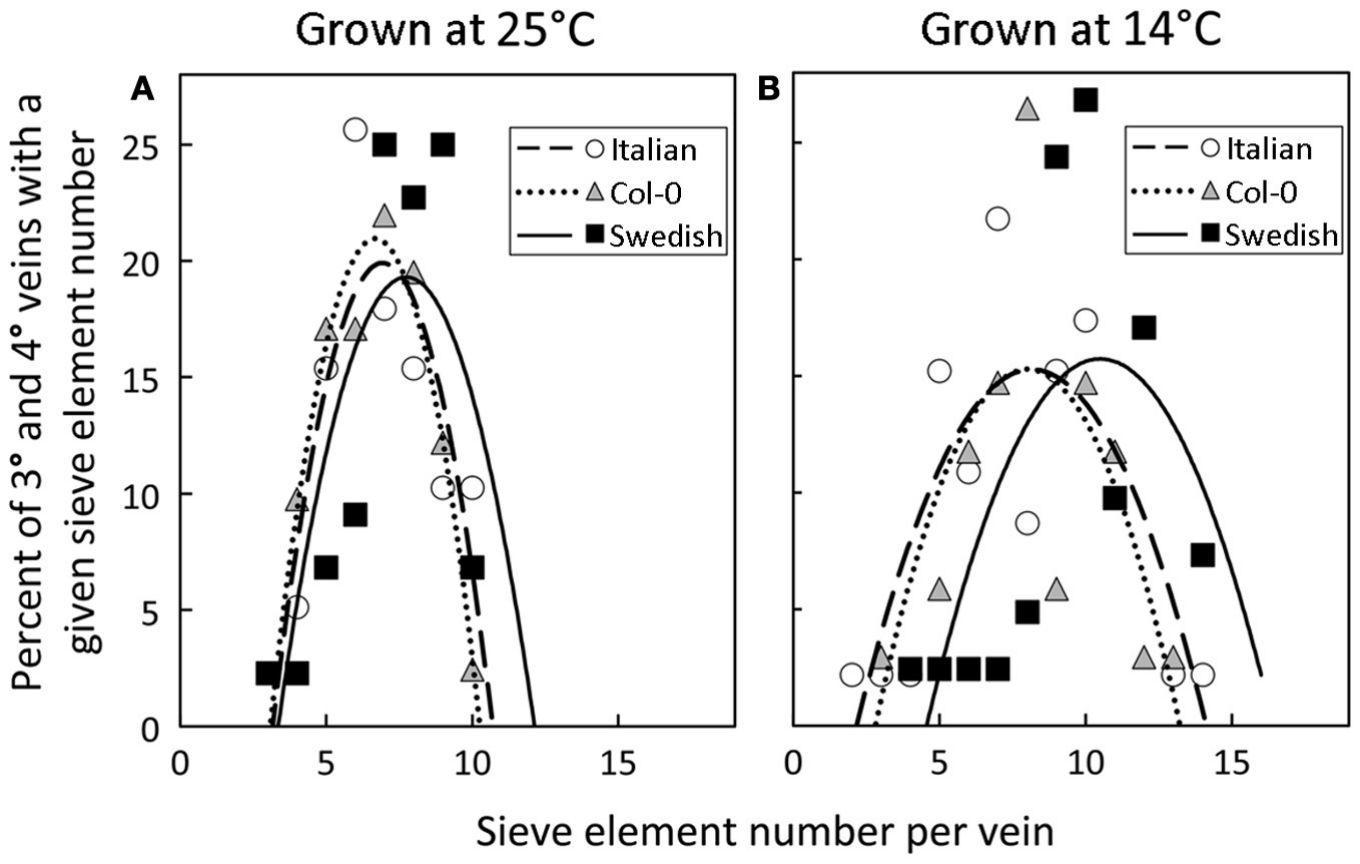
**Percent of third- and fourth-order veins (*y*-axis) with a given sieve element number per minor loading vein (*x*-axis) for Italian (open circles), Col-0 (gray triangles), and Swedish (black squares) *A. thaliana* ecotypes grown under moderate light (400 μmol photons m^−2^ s^−1^) in (A) warm (25°C) or (B) cool (14°C) conditions**. Second-order polynomial line fit for Italian (dashed line), Col-0 (dotted line), and Swedish (solid line) ecotypes.

A significant impact of temperature on vein development is revealed by examining the relative percentage of veins with distinct numbers of sieve elements in the three *A. thaliana* lines (Figure 8). The fourth- and third-order veins of all three ecotypes of *A. thaliana* grown at 25°C under either moderate or high light contained 10 or fewer sieve elements (Figure 3A), with a slightly greater mean sieve element number for the Swedish ecotype as represented by the second-order polynomial fit lines in Figure 8A. In contrast, when plants were grown at 14°C under moderate light (400 μmol photons m^−2^ s^−1^), some fourth- and third-order veins of all ecotypes contained more than 10 sieve elements, with the Swedish ecotype possessing the greatest number of sieve elements per minor vein (Figure 8B).

To evaluate the origin of the additional phloem cells that developed in the minor loading veins of leaves that grew under lower temperature and higher PFD, the total number of phloem cells and of xylem cells, as well as the ratio of phloem to xylem cells, were compared among the three ecotypes and four primary growth conditions. This comparison revealed that the total number of phloem cells increased with increased growth PFD, and especially with decreased growth temperature, whereas there was no consistent difference in the total number of xylem cells with growth condition (Table 1). The phloem to xylem cell ratio thus increased with increased PFD and decreased temperature during growth (Table 1). Additionally, relationships among the phloem cells of the minor loading veins were evaluated for leaves of the Swedish ecotype that developed under moderate light at the two temperatures. Whereas the number of companion cells per minor loading vein was significantly greater in the leaves that developed at the lower temperature, there was no significant difference in the number of companion cells per phloem cell or the number of companion cells per sieve element (Table 2).

**Table 1:** **The total number of phloem cells, the total number of xylem cells, and the ratio of phloem cell number to xylem cell number per minor loading (third- and fourth-order) vein in leaves of the Italian, Col-0, and Swedish ecotypes of *A. thaliana* grown under four different temperature and PFD regimes**.

**Ecotype**	**Vascular metric**	**Leaf temperature and PFD in μmol photons m^−2^ s^−1^ during growth**			
		**25°C, 400**	**25°C, 1000**	**14°C, 400**	**14°C, 1000**
Italian	No. of phloem cells	34 ± 1^b^	36 ± 2^a, b^	36 ± 1^a, b^	40 ± 2^a^
	No. of xylem cells	13 ± 1 n.s.	12 ± 1	12 ± 1	12 ± 1
	phloem/xylem	2.6	3	3	3.3
Col-0	No. of phloem cells	32 ± 1^b^	33 ± 1^b^	38 ± 3^b^	49 ± 2^a^
	No. of xylem cells	13 ± 1 n.s.	10 ± 1	10 ± 1	13 ± 1
	phloem/xylem	2.5	3.3	3.8	3.8
Swedish	No. of phloem cells	35 ± 1^b^	36 ± 1^b^	47 ± 1^a^	53 ± 2^a^
	No. of xylem cells	13 ± 1 n.s.	15 ± 1	13 ± 1	15 ± 1
	phloem/xylem	2.7	2.4	3.6	3.5

Means ± standard error of the mean (n = 4) and statistically significant differences indicated with lower case letters, i.e., means sharing a common letter are not statistically different from one another, P < 0.05, n.s., not significantly different. Statistics conducted independently for number of phloem and xylem cells per vein within each ecotype, i.e., comparisons conducted horizontally in the table.

**Table 2:** **Companion cell (CC) number, number of companion cells relative to all phloem cells, and number of companion cells per sieve element (SE) in the minor loading (third and fourth order) veins of leaves of the Swedish ecotype of *A. thaliana* that developed under moderate light (400 μmol photons m^−2^ s^−1^) at a leaf temperature of either 25°C or 14°C**.

**Leaf temperature during development**	**Number of companion cells**	**Number of CCs/phloem cells**	**Number of CCs per sieve element**
25°C	14.1 ± 0.7	0.41 ± 0.01	1.86 ± 0.08
14°C	19.4 ± 0.7	0.40 ± 0.01	1.93 ± 0.04
Level of significance	*P* < 0.001	n.s.	n.s.

Means ± standard error of the mean (n = 4), n.s., not significantly different.

**Figure 9:**
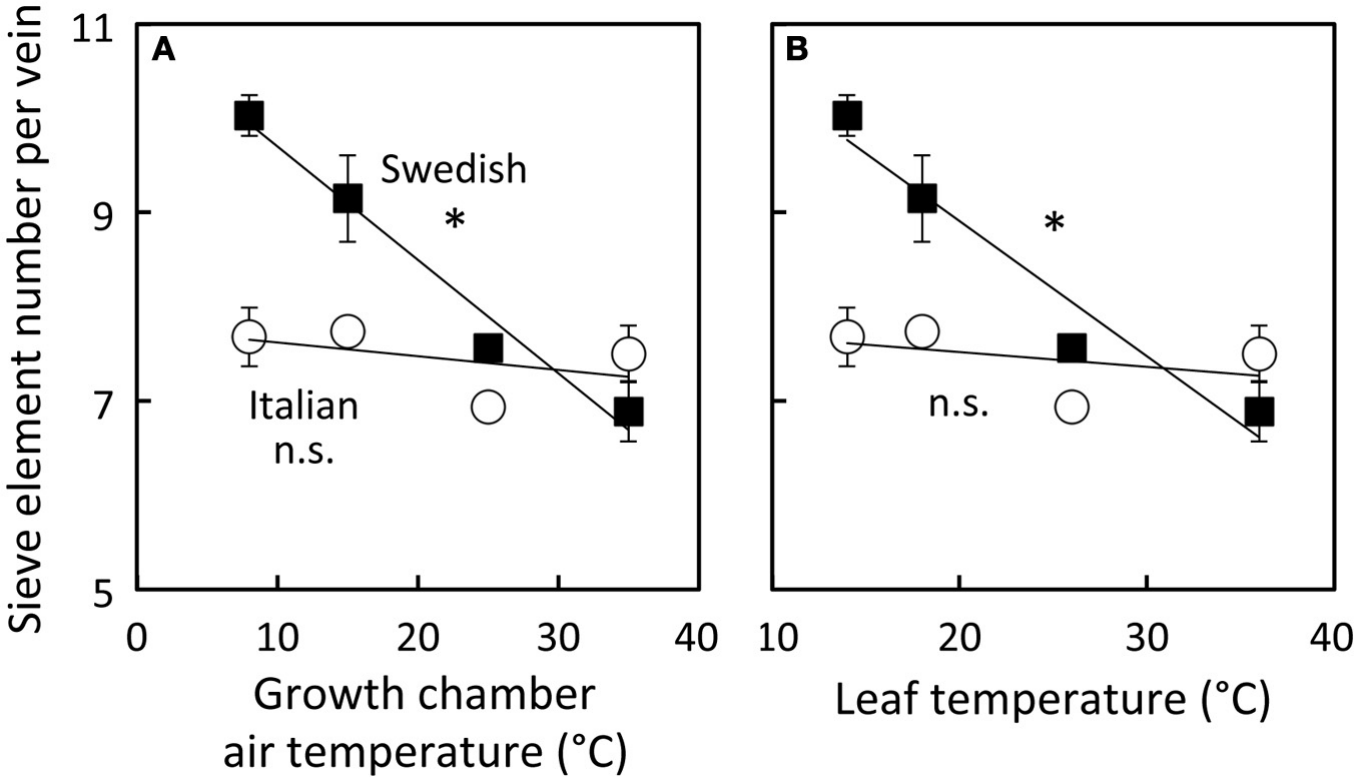
**Differences in sieve element number per minor loading (third- and fourth-order) vein among Italian (open circles) and Swedish (black squares) ecotypes of *A. thaliana* dependent on air temperature (A) or leaf temperature (B) during growth under moderate light (400 μmol photons m^−2^ s^−1^)**. Means ± standard error of the mean (*n* = 4) shown; significant relationship at *P* < 0.05 indicated by an ^*^. Linear regression analyses for each line are (**A**) Swedish *y* = −0.12 *x* + 11, *R*^2^ = 0.97 and Italian *y* = −0.01 *x* + 8, *R*^2^ = 0.22 and (**B**) Swedish *y* = −0.14 + 12, *R*^2^ = 0.94 and Italian *y* = −0.02 + 8, *R*^2^ = 0.17.

To examine how the number of sieve elements scaled with growth temperature under a common growth PFD condition, the number of sieve elements per minor loading vein was assessed from plants growing under four different growth temperature regimes under the 9 h photoperiod of moderate light (Figure 9). Sieve element number per minor vein increased linearly with decreasing temperature only slightly and not significantly in the Italian ecotype, but linearly and significantly (almost doubling from high to low growth temperature over the examined range) in the Swedish ecotype. The Col-0 ecotype exhibited a response that was intermediate between that of the Italian and Swedish ecotypes (data not shown).

## Discussion

We have demonstrated that the conditions under which plants are grown, and leaves have developed, affect the number of sieve elements, and overall phloem cross-sectional area, in minor loading (third- and fourth-order) veins of *A*. *thaliana*. Moreover, the magnitude of the impact of growing conditions on the phloem of minor loading veins depended on the geographic origin of the ecotypes of this species. On the other hand, the total number of xylem cells did not differ appreciably in the minor veins of leaves that developed under different growth conditions, and thus the additional phloem cells present in the minor veins of leaves that developed under high light, and particularly under low temperature, did not arise at the expense of xylem tissue. Furthermore, the development of additional phloem tissue into the plane of the vascular bundle normally occupied exclusively by xylem, and in extreme cases even above (adaxial to) the xylem, may be the most efficient way to increase phloem-loading capacity. Simply increasing the numbers of cells in the abaxial portion of the loading vein would increase the distance between the inner-most cells of the phloem and the bundle sheath cells, and those inner-most cells would likely receive considerably less sucrose that they might otherwise be able to load and transport. The sucrose synthesized in the photosynthetically active mesophyll cells of the leaves passes through the bundle sheath cells that surround the vascular tissue before being loaded into the phloem. By generating additional phloem cells in close proximity to the bundle sheath cells in the adaxial portion of the vein, and away from the other phloem cells, phloem loading should be maximized for the vein as a whole.

For an overwintering annual like *A*. *thaliana*, the importance of increasing the number of companion cells (as drivers of phloem loading) and sieve elements (as conduits for the flux of sugars) at cooler temperature may lie in the ability to keep sugars moving out of the leaf even as the viscosity of phloem sap increases with decreasing temperature. To further evaluate this possibility, the impact of decreasing temperature on the viscosity of sucrose, and the corresponding increase in sieve element numbers and cross-sectional area, were estimated. Deeken et al. (2002) reported that the concentration of sucrose in the phloem of *A. thaliana* was 0.34 ± 0.05 M, or approximately 12% (w/w). Swindells et al. (1958) determined that the viscosity of a 12% solution of pure sucrose was 1.25 centipoises at 25°C and increased to 1.66 centipoises when the temperature was lowered to 15°C. The ratio of the viscosity of a 12% sucrose solution at 15°C vs. 25°C is thus 1.33. The ratios of the numbers of sieve elements per minor loading vein (Figure 3A), and total sieve element cross-sectional area per minor loading vein (Figure 3B), from leaves that developed under low temperature (14°C leaf temperature) vs. warm temperature (25°C leaf temperature) but under common PFDs are shown in Table 3. These latter ratios are equal to, or greater than, the 1.33 ratio of the viscosity of a 12% sucrose solution at 15°C compared to 25°C for both the Swedish and Col-0 ecotypes, with the exception of the sieve element number in the foliar minor loading veins of the Col-0 ecotype grown under 400 μmol photons m^−2^ s^−1^ (Table 3). There are several factors that could contribute to deviations in these metrics, including the fact that the *A. thaliana* plants from which the phloem sucrose concentration was determined were only grown at moderate temperature and under lower PFDs than the plants characterized here (i.e., the concentration of sucrose in the phloem may be different in the plants grown in the current study) and the nocturnal temperature of the plants grown at the lower temperature in the current study dropped to 8°C for the duration of the night (when sucrose would have continued to be exported from the leaves). Nonetheless, the greater number of sieve elements, and particularly the sieve element cross-sectional area, of minor loading veins that developed under cooler temperature are likely to contribute to an increased loading and transport of sucrose in the minor veins of leaves that would otherwise not be possible. That the Swedish ecotype experiencing lower temperatures under natural conditions for a greater fraction of the year than the Italian ecotype (Ågren and Schemske, 2012) should show the greatest increase in, and greatest number of, phloem cells in response to growth at higher PFD and cooler temperature is consistent with the latter interpretation.

**Table 3:** **Ratio of total number of sieve elements per minor loading vein and of total sieve element cross-sectional area per minor loading vein from leaves that developed under low temperature (14°C leaf temperature) vs. under warm temperature (25°C leaf temperature)**.

**Ecotype**	**Growth PFD (μmol photons m^−2^ s^−1^)**	**Ratio from minor veins that developed at 14°C compared to 25°C**	
		**Sieve element number**	**Sieve element cross-sectional area**
Swedish	400	1.33	1.79
Swedish	1000	1.43	1.42
Col-0	400	1.22	1.53
Col-0	1000	1.41	1.51
Italian	400	1.11	1.10
Italian	1000	1.19	1.22

In a subsequent paper (Cohu et al., 2013, Under Review), we show that there is a strong and significant correlation between both number and cross-sectional area of the sieve elements and the cells associated with the sieve elements (companion and phloem parenchyma cells) and photosynthetic capacity in these three ecotypes of *A. thaliana* grown under different environmental conditions. The ability to increase the number of cells associated with loading and transporting sugars from the leaf is but one of several potential acclimatory responses leaves exhibit to presumably facilitate increased export of photosynthate from the leaves to accommodate higher rates of photosynthesis. Additional acclimatory responses include an increase in vein density (Amiard et al., 2005; Adams et al., 2007, 2013), an increase in the wall ingrowths of transfer cells in some apoplastic loaders (Amiard et al., 2005, 2007), and an increase in the volume of intermediary cells of symplastic loaders (Adams et al., 2013).

### Conflict of interest statement

The authors declare that the research was conducted in the absence of any commercial or financial relationships that could be construed as a potential conflict of interest.
